# Nursing Progress and Its Developments: The Local Executive Committee

**Published:** 1918-08-31

**Authors:** 


### THE MATRONS' AND SISTERS' DEPARTMENT.

### NURSING PROGRESS AND ITS DEVELOPMENTS.

### THE LOCAL EXECUTIVE COMMITTEE.

The basis on which the Local Centre is reared is the General Committee, representative of all nursing interests within the sphere of the Centre's activities, consisting mainly of members of the College of Nursing, but capable of including, if desired, others who may be interested in the movement, these latter having, however, no power to vote.

But if this is the groundwork or foundation of the College Centre, it is the Local Executive Committee which is responsible for building the house. On the quiet perseverance, sound initiative, and conciliatory character of its members the future of the Centre must depend. Experience has yet to show what number of members answers best for this committee. The College of Nursing has wisely left the Centres alone to frame their own rules. No doubt suggestions would be forthcoming from headquarters if appealed to, but there is no hard-and-fast stipulation that the Executive Committee must consist of a certain number of members, must meet at stated intervals, or transact such-and-such business. This liberty of action is clear gain to the budding branches. Their needs differ widely in different counties and so does their capability of carrying out concerted action. Cornwall and Manchester, for instance, cannot develop on the same lines. Absolute freedom to organise as may be best under local conditions appears to be the first preliminary to success.

First among the qualities needed for building up a strong and united nucleus of nurses combining for professional purposes is the gift of doggedness. True for all enterprises, the motto "It's dogged that does it," applies with special force to committees which set out to guide their fellow-workers in the path of unity. For the first response may be poor. Politicians are not the only persons who like sitting on the fence. Yet an appeal quietly renewed at intervals comes home at last. Or the first enthusiasm may kindle more nurses into acquiescence than are found to be willing to perform their promises. Still, the steady pressure of regular notification, the attitude of quiet reliance on the good will of all concerned, must be kept up through every discouragement. The machine must go on revolving apparently unconcerned with the greater or less amount of material with which it deals. And in good time, by regular meetings, regardless of some poor attendances, by punctual reminders, regardless of the irresponsive air of many over-busy members, the committee will win the confidence of the whole body.

But though we put the art of keeping things going regularly as of first importance in the building of the Centre, we would not be thought to overlook the value, the irreplaceable value, of ideas in its development. Initiative is all-important: not the initiative which dashes at a dozen attractive schemes and puts not one into working order; but the initiative which seizes upon the local requirements, is deterred by no difficulties, and plants success where the objector sees nothing but failure. From the point of view of initiative, of actually getting things accomplished, it may be held that the smaller the committee the better. Yet it is well for eager minds to be forced to think out in detail and submit to cooler heads the plans which are to ripen for the good of all. Only by the blending of the practical with the ideal can big things be brought into existence, and the two are not always present in the same individual.

And this brings us to the third preliminary to success in the conduct of the Executive Committee. It must be conciliatory, or its work will from the outset be terribly, perhaps irretrievably, marred. The essence of sitting in committee is give and take. Under no other conditions does human nature betray itself more candidly. Many persons who have not previously convicted themselves of possessing a "temper" (meaning the wrong kind of temper) are wont to be moved when they find themselves misunderstood or outvoted in some darling project. The impulse to resign when things go contrary to the individual judgment is strong at such times, but examined in the light of reason it is seen to be unworthy and directly subversive of that government by the majority which decides all our affairs, from the Cabinet down to the Village Nursing Association. If the Local Executives are to bring their work to perfection they will need to the full the spirit of conciliation which refuses to impute unworthy motives, which can bear defeat, learn without impatience to hear both sides of a question, and submit good-humouredly to pin-pricks from the irritable. The nursing world has suffered so much from heated debates that the object-lesson of Nursing Committees working harmoniously throughout the country would refresh the soul like draughts of spring water.

